# Mechanically Flexible Thermoelectric Hybrid Thin Films by Introduction of PEDOT:PSS in Nanoporous Ca_3_Co_4_O_9_

**DOI:** 10.1021/acsomega.2c02875

**Published:** 2022-06-28

**Authors:** Binbin Xin, Lei Wang, Arnaud Le Febvrier, Anna Elsukova, Biplab Paul, Niclas Solin, Per Eklund

**Affiliations:** † Thin Film Physics Division, Department of Physics, Chemistry and Biology (IFM), Linköping University, SE-58183 Linköping, Sweden; ‡ Electronic and Photonic Materials Division, Department of Physics, Chemistry and Biology (IFM), Linköping University, SE-58183 Linköping, Sweden

## Abstract

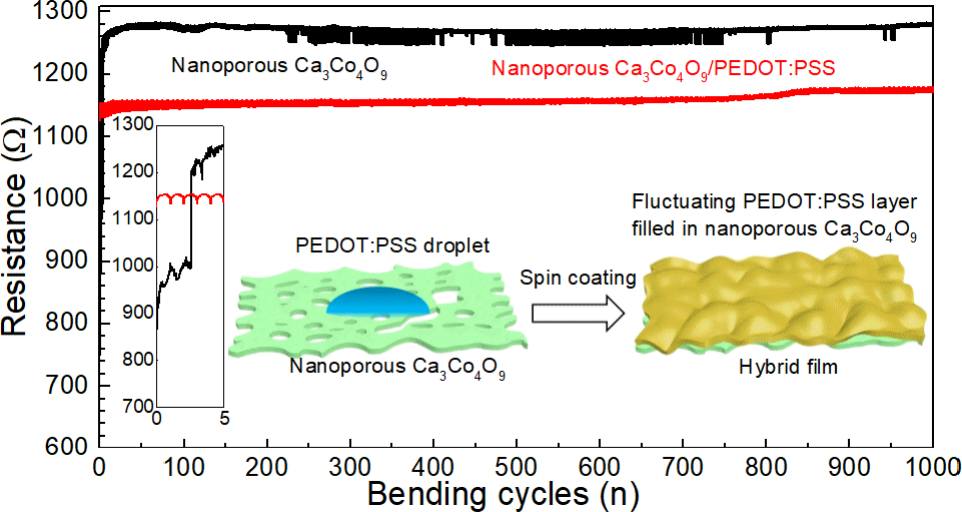

Nanoporous Ca_3_Co_4_O_9_ exhibits high thermoelectric properties and low thermal conductivity and can be made mechanically flexible by nanostructural design. To improve the mechanical flexibility with retained thermoelectric properties near room temperature, however, it is desirable to incorporate an organic filler in this nanoporous inorganic matrix material. Here, double-layer nanoporous Ca_3_Co_4_O_9_/PEDOT:PSS thin films were synthesized by spin-coating PEDOT:PSS into the nanopores. The obtained hybrid films exhibit high Seebeck coefficient (∼+130 μV/K) and thermoelectric power factor (0.75 μW cm^–1^ K^–2^) at room temperature with no deterioration in electrical properties after cyclic bending tests (98% preservation of electrical conductivity after 1000 cycles bending to a bending radius of 3 mm). Compared with the nanoporous Ca_3_Co_4_O_9_ thin film, the mechanical flexibility of the hybrid film can be effectively improved after hybrid with PEDOT:PSS with only a slight decrease of the thermoelectric properties.

## 1 Introduction

Calcium cobaltate Ca_3_Co_4_O_9_ has a complex crystal structure composed of CoO_2_ conductive layers and rock-salt type Ca_2_CoO_3_ insulating layers. This material is an attractive p-type thermoelectric material with high Seebeck coefficient S, moderate electrical conductivity σ and low thermal conductivity κ. Single crystals of Ca_3_Co_4_O_9_ show a reasonably high thermoelectric figure of merit ZT (ZT = *S*^2^σ*T*κ^–1^, where *T* is the working temperature) value of around 0.87 at 973 K.^1^ The orientation of this layered ceramic is also important for the thermoelectric properties,^2,3^ offering a possibility for tailoring the thermoelectric properties in thin films.^4−8^ Ca_3_Co_4_O_9_ also has the typical advantages of oxides, including the chemical stability at high temperatures.^9^

With the development of portable or wearable electronic devices, maintenance-free thin film thermoelectrics without recharging is being investigated as possible power supply.^10^ Ca_3_Co_4_O_9_ films typically show high thermoelectric power factor *P* (*P = S*^2^σ) (∼0.5 μW cm^–1^ K^–2^ at room temperature and ∼2.7 μW cm^–1^ K^–2^ at 720 K).^11^ Growing films on common inorganic substrates such as silicon,^12^ SrTiO_3_,^4^ and sapphire^5,11^ restrict the formation of film–substrate systems with mechanical flexibility. Fully inorganic flexible Ca_3_Co_4_O_9_ films can be obtained by nanostructuring and porosity^6,13^ and growing on flexible inorganic substrates.^14,15^ We have previously showed that annealing CaO/Co_3_O_4_ multilayers yields nanograined and porous Ca_3_Co_4_O_9_ films with power factors as high as 1 μW cm^–1^ K^–2^ at room temperature.^7,8,16^ Textured nanograins and faceted nanopores alleviate the brittleness and induce mechanical flexibility. Nevertheless, the mechanical flexibility is still lower compared to those of organic polymer thermoelectric materials. Poly(3,4-ethylenedioxythiophene) polystyrenesulfonate (PEDOT:PSS) is one of the most promising p-type thermoelectric materials for flexible thermoelectric application because of its high electrical conductivity, low thermal conductivity, excellent stability, flexibility, and commercial availability.^17−19^ As-prepared films made directly from the aqueous PEDOT:PSS dispersion usually have very low S (15–18 μV K^–1^)and σ (0.2–1 S cm^–1^).^20^ The thermoelectric properties of PEDOT:PSS films can be improved by doping or secondary doping, which leads to improvements in *S* and σ.^21^ However, the inherently moderate thermoelectric properties of polymers compared to many inorganic materials, presents a strong motivation for the development of hybrid inorganic/organic materials for mechanically flexible thermoelectrics. In such an approach, a mechanically flexible thermoelectric polymer can be filled with conventional inorganic thermoelectric materials in order to realize a hybrid structure combining mechanical flexibility with high thermoelectric performance.^22−24^ However, in practice when combining oxides with PEDOT:PSS, the results are a considerable decrease in power factor, mainly due to a reduction of the Seebeck coefficient.^25−28^

Here, we use PEDOT:PSS as filler in nanoporous Ca_3_Co_4_O_9_ to form double-layer inorganic/organic hybrid by spin coating. Compared with nanoporous Ca_3_Co_4_O_9_ films, the Seebeck coefficient of these hybrid films remains high at ∼+130 μV/K with a power factor of ∼0.75 μWcm^–1^K^–2^ at room temperature. The electrical properties are fully retained after cyclic bending tests with 98% preservation of electrical conductivity after 1000 cycles bending to a bending radius of 3 mm. Therefore, the mechanical flexibility can be effectively improved by forming a hybrid with PEDOT:PSS with only a small influence on thermoelectric properties compared with nanoporous Ca_3_Co_4_O_9_ thin film.

## 2 Experimental Section

### 2.1 Synthesis

Nanoporous Ca_3_Co_4_O_9_ thin films were synthesized by annealing Ca(OH)_2_/Co_3_O_4_ multilayer film deposited by magnetron sputtering onto mica substrates. The detailed procedure is described in our previous work.^16^ The different solid content (0.25, 0.5, and 1 wt % (in water)) of PEDOT:PSS dispersions can be prepared based on raw PEDOT:PSS (Clevios PH1000). The PEDOT:PSS dispersions with different solid content were deposited by spin-coating (2000 rpm for 40 s) onto the nanoporous Ca_3_Co_4_O_9_ thin films. The resulting wet films were heated to 120 °C to remove residual solvent (water) to form PEDOT:PSS film. The double-layer inorganic/organic hybrid films were prepared with different thicknesses of the PEDOT:PSS layer. The pure PEDOT:PSS films were prepared by spin-coating the raw PEDOT:PSS aqueous solution on glass substrates of 1.3 × 1.3 cm^2^ with the same conditions. The ethylene glycol (EG) treatment was performed by dropping 100 μL EG solution on the PEDOT:PSS film or hybrid film at 120 °C. Then the treated films were dried, rinsed with DI water three times, and dried again.

### 2.2 Characterization

X-ray diffraction (XRD) measurements were performed using a X’Pert PRO MRD diffractometer from PANalytical using Cu Kα_1,2_ radiation with a nickel filter in Bragg–Brentano configuration (θ–2θ scans). The surface morphology and pore structure of the films were studied by scanning electron microscopy (SEM) using a LEO Gemini 1550 Zeiss with a 10 kV operating voltage. Transmission electron microscopy (TEM) was carried out using a FEI Tecnai G2 TF20 UT instrument operated at 200 kV. A nanoporous Ca_3_Co_4_O_9_ sample suitable for TEM measurements was prepared by mechanically polishing face-to-face glued sandwiches of two sample pieces mounted on a Ti grid down to a thickness of 50 μm; the sample was then ion-milled with 2–5 kVAr^+^ beams incident at 5° in a Gatan precision ion polishing system. The static contact angles (CA) were measured using the sessile drop of 4 μL of various concentrations of PEDOT:PSS diluted by DI water with a CAM200 optical contact angle meter (KVS Instrument, Finland).The electrical conductivity σ was calculated from the sheet resistance measured with a four-point probe Jandel RM3000 station, using the thicknesses of the films determined from cross-sectional SEM images. For the hybrid films, the thickness used for the calculation of σ is the total thickness of the polymer layer and the nanoporous Ca_3_Co_4_O_9_ layer. The Seebeck coefficient α was measured in an in-house setup, as in our earlier work.^29^ The mechanical flexibility was evaluated by measuring the change in the two-point probe resistance (Δ*R*) during 1000 cycling. The bending tests were performed by clamping between two electrodes which can be translated with precision using a step motor. The Cu electrodes were connected to a Keithley 2001 multimeter to measure the resistance collected automatically using a homemade LabVIEW script. To ensure a minimum contact resistance, a silver paste line is placed between the sample and the electrode before being clamped. The linear electric motor can set the moving distance, bending cycles, and rates. The repeated bending test was carried out 1000 times with a bending radius of 3 mm at the rate of 0.1 mm s^–1^ over 8 mm length sample.

## 3 Results and Discussion

**Figure 1:**
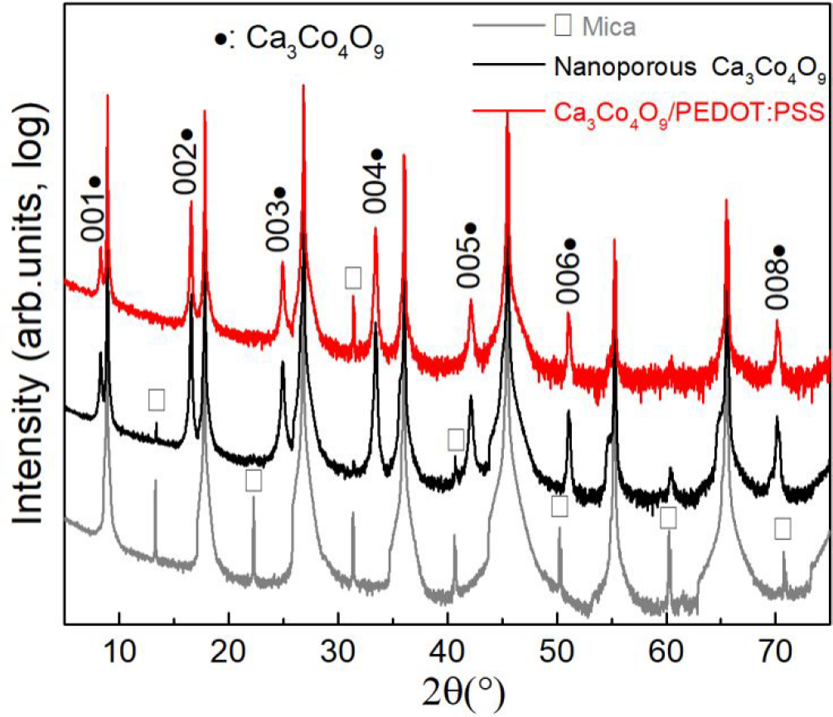
X-ray diffractograms of the mica substrate, the nanoporous Ca_3_Co_4_O_9_ growing on mica, and nanoporous Ca_3_Co_4_O_9_ hybrid with PEDOT:PSS.

Figure 1 shows X-ray diffraction patterns of the mica substrate, a Ca_3_Co_4_O_9_ film on mica, and Ca_3_Co_4_O_9_ hybrid with PEDOT:PSS on mica substrate. In addition to the substrate peaks, the diffraction peaks observed for both the pristine sample and the hybrid film are at 2θ = 8.32°, 16.58°, 24.95°, 33.44°, 42.16°, 51.07°, 60.45° and 70.14° (not listing the peaks of the mica substrate). These peaks are identified as 00l reflections from Ca_3_Co_4_O_9_ (ICDD file 00-023-0110). Therefore, the Ca_3_Co_4_O_9_ films have textured basal planes oriented parallel to the substrate surface. It is important that Ca_3_Co_4_O_9_ retains the structure after hybridizing to make sure there are high thermoelectric properties in hybrid film. It is important to note that PEDOT:PSS (PH1000) dispersions are acidic with typical pH values about 1.5–2.5 (25 °C).^30^ These acidic conditions could potentially lead to etching of the inorganic component. However, after spin coating and heating at 120 °C for about 20 min, we find that the diffraction pattern of the Ca_3_Co_4_O_9_/PEDOT:PSS film matches with that of Ca_3_Co_4_O_9_, and the intensities are almost identical compared with those of nanoporous Ca_3_Co_4_O_9_ film. This result demonstrates that the acidic conditions during spin coating and drying does not etch and decompose the Ca_3_Co_4_O_9_ structure. The peaks of PEDOT:PSS cannot be observed in Figure 1 due to their low intensity. The XRD pattern of PEDOT:PSS film only on glass and board peak at ∼25° and can be seen in Figure S1, which indicates the amorphous structure of PEDOT:PSS.

**Figure 2:**
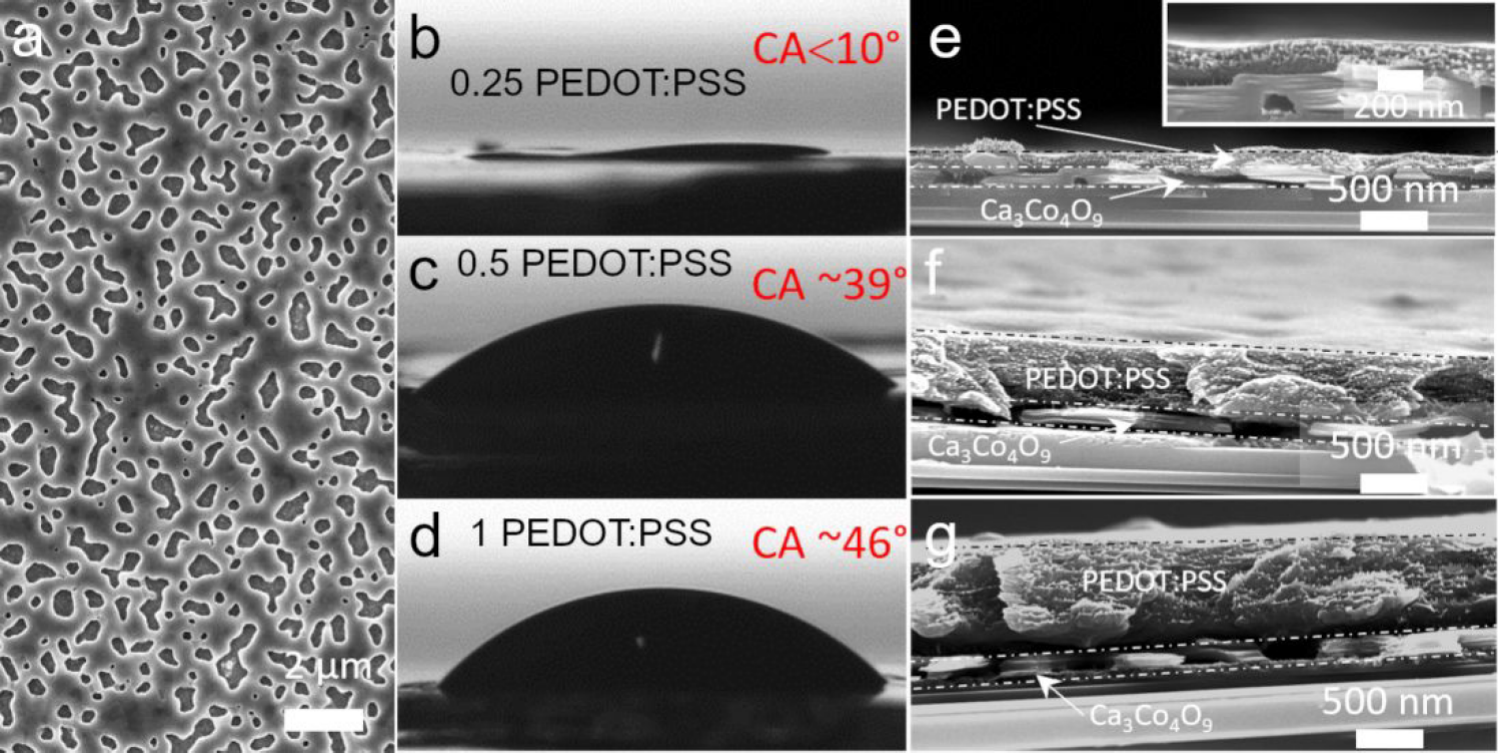
(a) SEM micrograph of the nanoporous Ca_3_Co_4_O_9_ films; the contact angle images (b–d) of PEDOT:PSS dispersions dropping on nanoporous Ca_3_Co_4_O_9_ film and the cross-sectional SEM images (e–g) of Ca_3_Co_4_O_9_/PEDOT:PSS hybrid films with different solid content 0.25, 0.5, and 1 wt % of PEDOT:PSS dispersions.

A typical SEM image of the morphology of a Ca_3_Co_4_O_9_ film is shown in Figure 2a. The film is composed of a matrix with an irregular nanopore structure with an average pore size of 206 ± 100 nm (Figure 2a) and nanopore size distribution in the range of 0–600 nm (Figure S3). Cross-sectional TEM micrographs (Figure S2a) reveal that the film is composed of a nanoporous crystalline Ca_3_Co_4_O_9_ layer with an apparent thickness about 200 nm on top of an amorphous layer. The nanopore with a depth of 200 nm is located in the crystalline layer and not in the amorphous layer. HRTEM reveals the high crystal quality of the films with an SAED pattern (Figure S2b) confirming that the (001) basal planes are oriented parallel to the film surface, matching the XRD results described above (Figure 1).

The different solid content 0.25, 0.5, and 1 wt % of PEDOT:PSS dispersions were dropped on nanoporous Ca_3_Co_4_O_9_ film to measure the contact angle when the droplet was stable (Figure 2b–d). The CA increases from less than 10° to 46° with increasing the solid content of PEDOT:PSS dispersions, indicating the better wetting behavior of lower concentration. After drying, the PEDOT:PSS layer was forming on the top of Ca_3_Co_4_O_9_ film and the apparent thickness in the hybrid film was ∼200, ∼500, and ∼990 nm with increasing the concentration, respectively (Figure 2e–g). The layered structure of Ca_3_Co_4_O_9_ can be observed in the hybrid films from the cross-sectional SEM images (Figure 2e–g), which demonstrates that the acidity of the PEDOT:PSS dispersion did not cause any significant damage to the Ca_3_Co_4_O_9_ layered structure. In addition, when the cross-sectional SEM image is tilted at about 20° (Figure 2h), a PEDOT:PSS layer with fluctuating thickness can be observed with a partly depressed polymer layer above Ca_3_Co_4_O_9_ nanopores.

**Figure 3:**
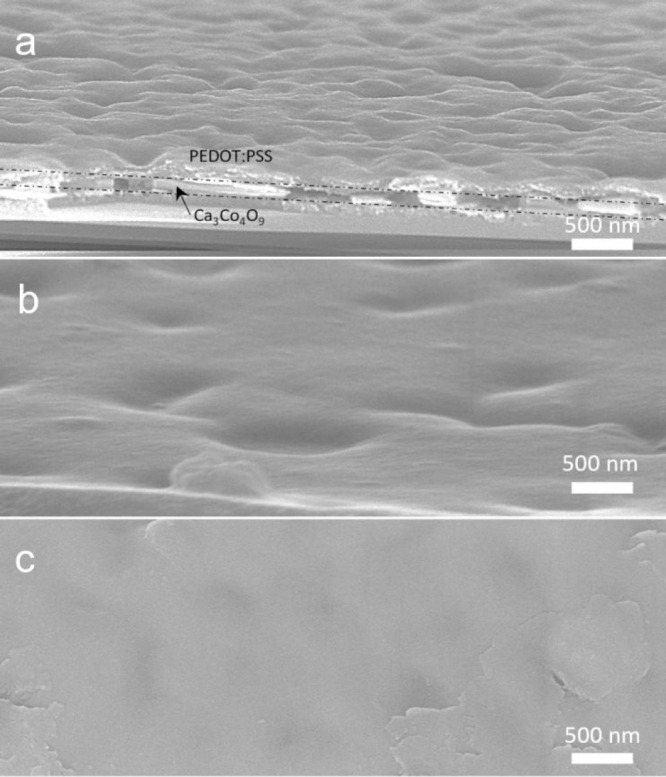
Cross-sectional SEM images (a–c) with tilting 20° of Ca_3_Co_4_O_9_/PEDOT:PSS hybrid films with different solid content 0.25, 0.5, and 1 wt % of PEDOT:PSS dispersions.

**Figure 4:**
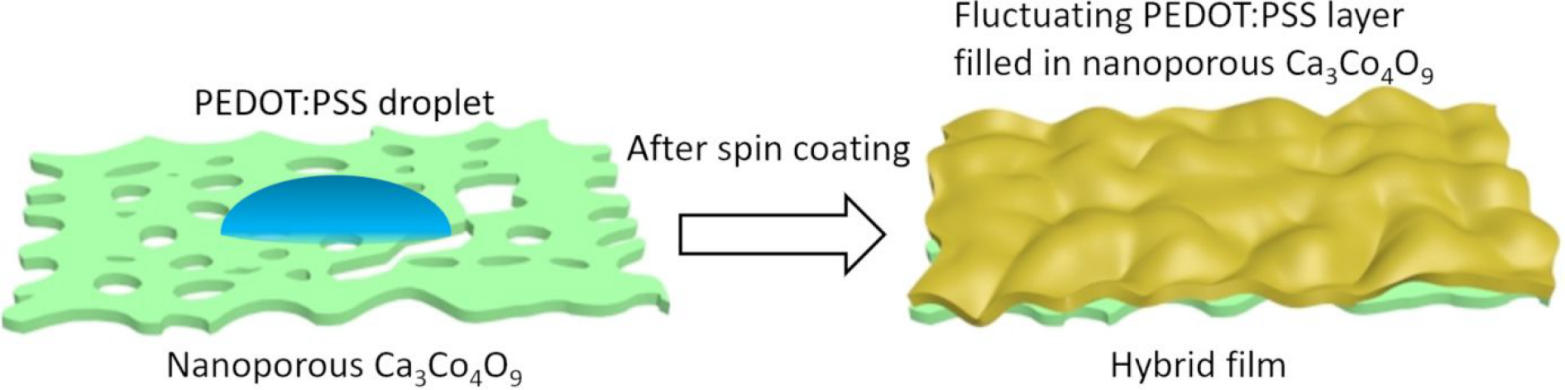
Schematic illustration of the formation process of the hybrid film with low concentration PEDOT:PSS (0.25).

With an increasing thickness of PEDOT:PSS, the top surface changes from uneven to smooth (Figure 3a–c). The PEDOT:PSS solution with low concentration (0.25) can fill in nanopores to form fluctuating polymer layer embracing nanoporous Ca_3_Co_4_O_9_ hybrid film by spin coating, as showing in Figure 4. The reason is that the lower concentration PEDOT:PSS solution has better wetting behavior and can effectively fill in the nanoporous Ca_3_Co_4_O_9_ film.

**Figure 5:**
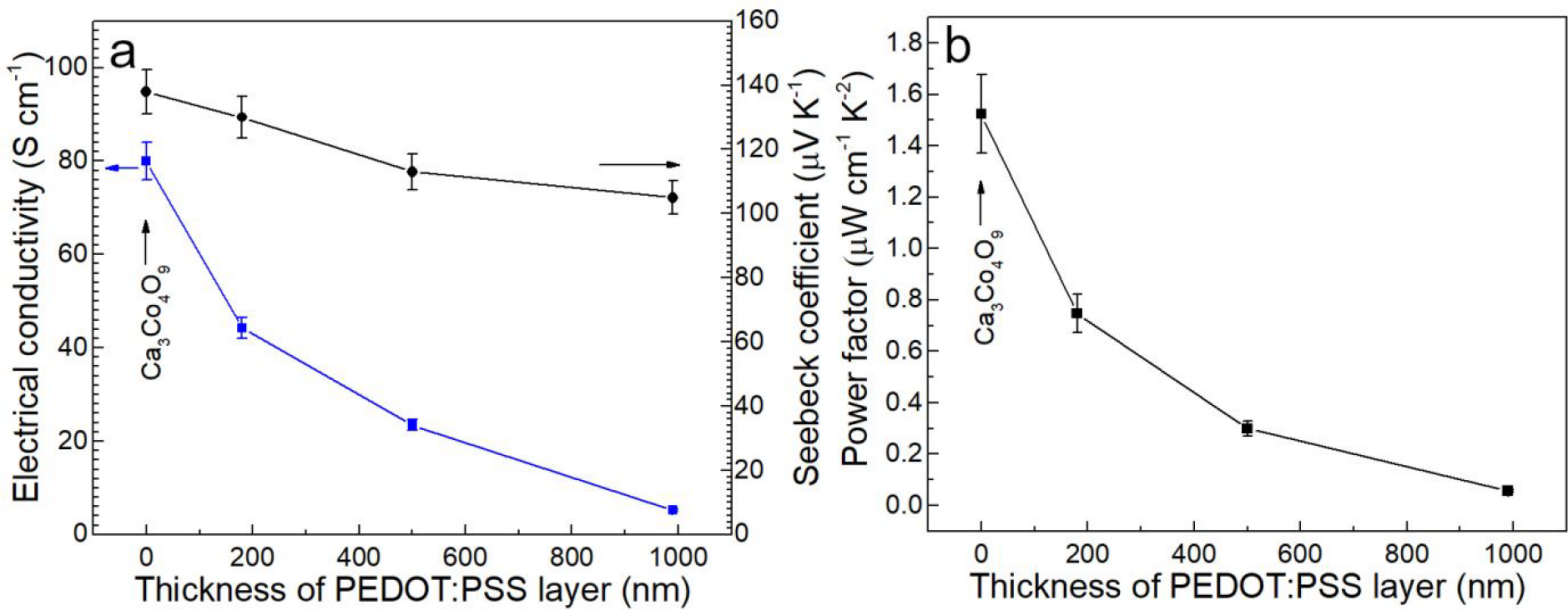
(a) Seebeck coefficient and electrical conductivity and (b) power factor of nanoporous Ca_3_Co_4_O_9_ thin film and Ca_3_Co_4_O_9_/PEDOT:PSS films at room temperature.

Figure 5 presents the thermoelectric properties of Ca_3_Co_4_O_9_ thin film (without any PEDOT:PSS) and Ca_3_Co_4_O_9_/ PEDOT:PSS hybrid films. Regarding the Seebeck coefficient, *S* increases from 105 up to 130 μV/K when decreasing the thickness of PEDOT:PSS layer in hybrid films at room temperature (Figure 5a). *S* of hybrid films are between the value of PEDOT:PSS (22 μV/K) and Ca_3_Co_4_O_9_ (135 μV/K). Similarly, the variations in σ are in the range from 5 to 44 S cm^–1^ (with higher conductivity being obtained when decreasing the fraction of PEDOT:PSS in hybrid films), which lies between the value of pure PEDOT:PSS (∼1 S cm^–1^) and Ca_3_Co_4_O_9_ (80 S cm^–1^). The reason for the observed trends may be that the double-layer inorganic/organic structure, having nearly a parallel structure, leads to that the thermoelectric properties have a trend changing based on parallel model with the volume fraction of polymer^31^
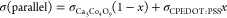
1
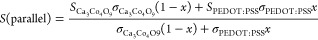
2where σ(parallel) and *S*(parallel) are the calculated electrical conductivity and Seebeck coefficient of the hybrids based on the parallel connected mode, and *x* is the volume fraction of the PEDOT:PSS in the hybrids. For the hybrid film with lowest content of polymer, the power factor is 0.75 μW cm^–1^ K^–2^, which is half of the value of nanoporous Ca_3_Co_4_O_9_ (1.5 μW cm^–1^ K^–2^) at room temperature (Figure 5b).

That PEDOT:PSS is a mixture of electronically conductive PEDOT and electronically insulating PSS, and the properties of PEDOT:PSS can be modified by solvent treatment. During solvent treatment (in this study, EG is employed as the solvent), the aim is to selectively remove PSS, which may lead to an improved electronic conductivity of the PEDOT:PSS material^21,32^ and hence also a modification of the thermoelectric properties. The thermoelectric properties of the PEDOT:PSS film and hybrid treated with EG are listed in Table 1. The σ of the EG treated PEDOT:PSS film is 200 S cm^–1^, which is much higher than that of Ca_3_Co_4_O_9_. For the hybrid film with 200 nm of PEDOT:PSS layer, the σ of the hybrid material increases from 44 S cm^–1^ to 87 S cm^–1^ and *S* decreases from 130 to 70 μV/K after EG treated. Hence, the power factor of the hybrid film becomes lower (0.43 μW cm^–1^ K^–2^) after EG treatment.

**Table 1:** Thermoelectric Properties of the Pristine PEDOT:PSS film, Ca_3_Co_4_O_9_ Film, and PEDOT:PSS Film and Hybrid Treated with Ethylene Glycol (EG)

	electrical conductivity (S cm^–1^)	seebeck coefficient (μV K^–1^)	power factor (μW cm^–1^ K^–2^)
Ca_3_Co_4_O_9_ film	80 ± 4	135 ± 6	1.5 ± 0.15
hybrid film (200 nm PEDOT:PSS)	44 ± 2.2	130 ± 6	0.75 ± 0.07
pristine PEDOT:PSS	∼1 ± 0.05	22 ± 1.1	0.00048 ± 0.00005
EG treated PEDOT:PSS	200 ± 10	21 ± 1.1	0.088 ± 0.009
EG treated hybrid film (200 nm PEDOT:PSS)	87 ± 4	70 ± 3.5	0.43 ± 0.04

**Figure 6:**
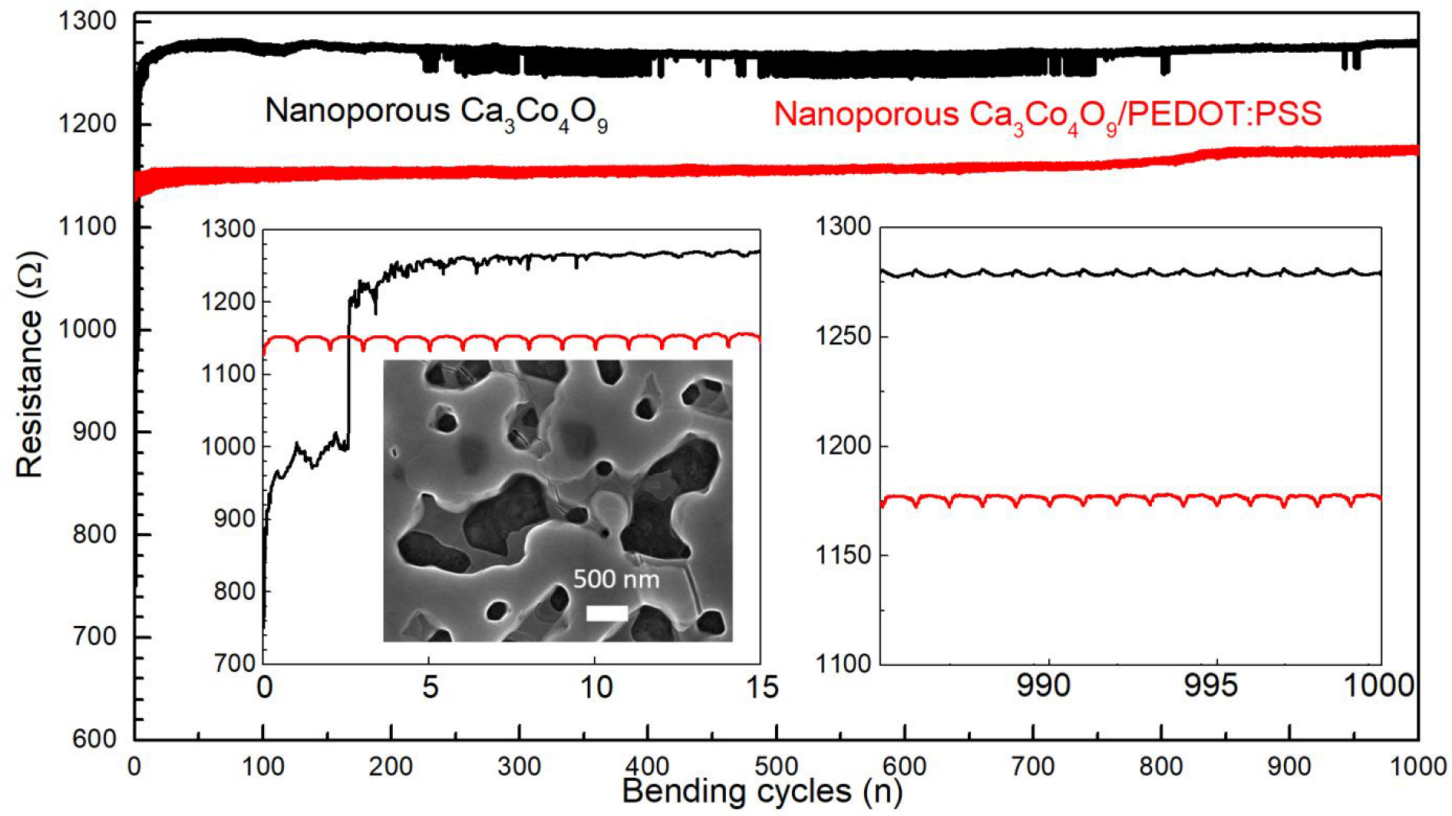
Mechanical bending measurement of nanoporous Ca_3_Co_4_O_9_ thin film and Ca_3_Co_4_O_9_/PEDOT:PSS film with the thickness (200 nm) of Ca_3_Co_4_O_9_ layer before EG treatment; the inset SEM image of nanopores Ca_3_Co_4_O_9_ thin film after bending measurement.

The bending flexibilities of inorganic nanoporous Ca_3_Co_4_O_9_ thin film and hybrid film with the thickness (200 nm) without EG treatment were evaluated by measuring the change in the two-point resistance Δ*R* for a number of bending cycles. Both films received identical mechanical treatment while measuring the resistance of the material during 1000 cycles. The electrical resistance of the nanoporous Ca_3_Co_4_O_9_ film increases by a factor 2 after the first 5 cycles (Figure 6). The SEM images taken after the 5 bending cycles reveal the presence of cracks, which leads to a sudden increase of the electrical resistance measured. Nevertheless, after the drop during the first 5 cycles, the resistance did not undergo any dramatic changes for up to 1000 bending cycles (Figure 6). In this type of experiment, we find that the presence of PEDOT:PSS in pores leads to a dramatic improvement in performance. In contrast with the nanoporous material (without PEDOT:PSS), the hybrid film stays relatively constant from the beginning until the end of the 1000 cycles with a resistance around 1172 Ohms. The PEDOT:PSS filling in the nanopores Ca_3_Co_4_O_9_ can accordingly effectively protect the weak neck part of the nanoporous film to preventing breakage during bending. Then the Seebeck coefficient and electrical conductivity of these film were measured after 1000 bending cycles (Table S1). After bending, their Seebeck coefficient almost did not change and electrical conductivity reduced around 20%, which were measured by four-point probe. Taking both the thermoelectric characteristics and mechanical performance, the Ca_3_Co_4_O_9_/PEDOT:PSS hybrid shows a significantly improved mechanical flexibility coupled with only a modest decrease the Seebeck coefficient and electrical conductivity, compared to the nanoporous Ca_3_Co_4_O_9_ film.

## 4 Conclusion

Double-layer nanoporous Ca_3_Co_4_O_9_ /PEDOT:PSS thin film can be obtained by filled in PEDOT:PSS into the nanopores by spin coating. The optimum hybrid film has a higher Seebeck coefficient (around 130 μV/K) than PEDOT:PSS and power factor (0.75 μW cm^–1^ K^–2^) at room temperature. There is no deterioration in electrical conductivity and Seebeck coefficient after 1000 cyclic bending tests with a bending radius of 3 mm for the hybrid film. The mechanical flexibility of the hybrid film can be effectively improved by filling fluctuating PEDOT:PSS layer in the nanoporous Ca_3_Co_4_O_9_.

## Supporting Information Available

Additional XRD data of PEDOT:PSS on glass, cross-sectional transmission electron micrograph images, nanopore size distributions determined from analyses of SEM micrographs from the Ca_3_Co_4_O_9_ films, and thermoelectric properties of the Ca_3_Co_4_O_9_ film, and hybrid film before and after 1000 cyclic bending tests (PDF)
