# Supplementary material for: Mechanically Flexible Thermoelectric Hybrid Thin Films by Introduction of PEDOT:PSS in Nanoporous Ca3Co4O9
Source: ACS Omega. 2022 Jun 28;7(27):23988–94. doi: 10.1021/acsomega.2c02875 (PMC9281307; doi:10.1021/acsomega.2c02875)
Supplement: Supplementary file 1 — ao2c02875_si_001.pdf [file ao2c02875_si_001.pdf]

Supporting Information

Mechanically flexible thermoelectric hybrid thin films by introduction of PEDOT: PSS in nanoporous $Ca_3Co_4O_9$

Binbin Xin [a,], Lei Wang [b], Arnaud Le Febvrier [a], Anna Elsukova [a], Biplab Paul [a], Niclas Solin [b], and Per Eklund [a,*]*

a, Thin Film Physics Division, Department of Physics, Chemistry and Biology (IFM), Linköping University, SE-58183 Linköping, Sweden

b, Electronic and Photonic Materials Division, Department of Physics, Chemistry and Biology (IFM), Linköping University, SE-58183 Linköping, Sweden

* Corresponding authors. Email: binbin.xin@liu.se; per.eklund@liu.se

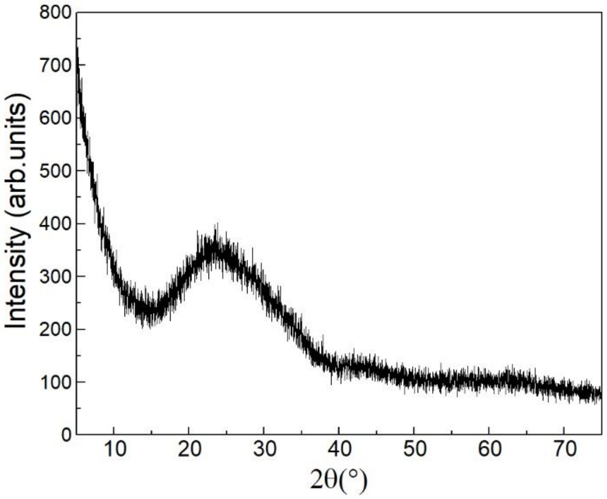

Figure S1. The XRD pattern of PEDOT:PSS film on glass.

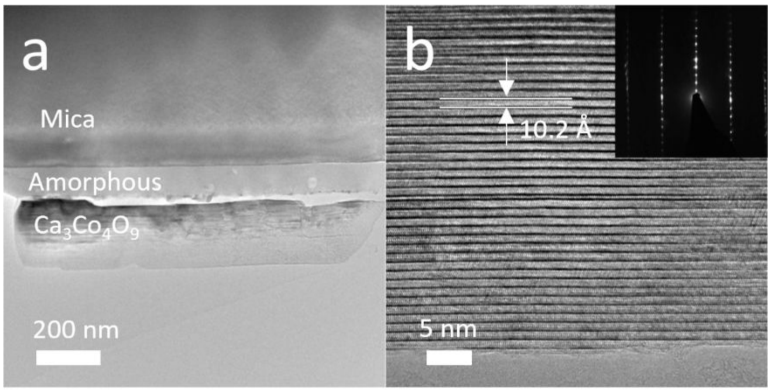

Figure S2. (a) Cross-sectional low-magnification transmission electron micrograph and (b) Lattice-resolved image of the crystalline $Ca_3Co_4O_9$ layer with corresponding SAED patterns.

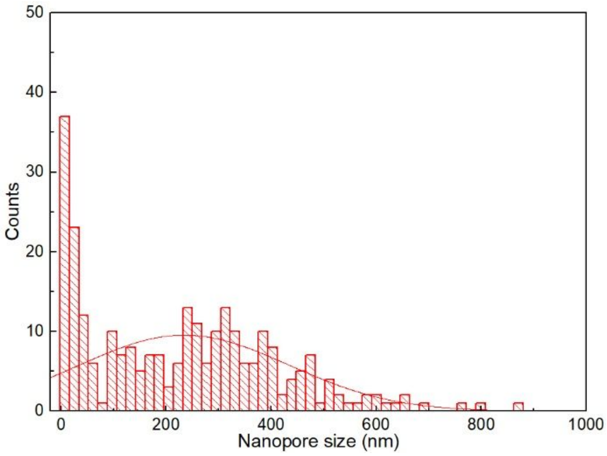

Figure S3. Nanopore size distribution obtained from analyses of SEM micrographs from nanoporous $Ca_3Co_4O_9$ film obtained from Figure 2a.

Table S1. Thermoelectric properties of the $Ca_3Co_4O_9$ film, and hybrid film before and after 1000 cyclic bending tests.

	Electrical conductivity (S cm^{-1})	Seebeck coefficient (μV K^{-1})
$Ca_3Co_4O_9$ film	80±4	135±6.5
$Ca_3Co_4O_9$ film after bending	31±5	135±6.5
Hybrid film (200 nm PEDOT:PSS)	44±2.2	130±6
Hybrid film after bending	35±5	123±6
